# A Systematic Review of Heated Intrathoracic Chemotherapy for Thymic Epithelial Tumors and the First Case Report of a Robotic Approach: Could a Minimally Invasive Approach Offer a New Paradigm of Care?

**DOI:** 10.3390/jcm14124094

**Published:** 2025-06-10

**Authors:** Russell Seth Martins, Elizabeth Christophel, Kostantinos Poulikidis, Syed Shahzad Razi, M. Jawad Latif, Jeffrey Luo, Faiz Y. Bhora

**Affiliations:** 1 Division of Thoracic Surgery, Department of Surgery, Hackensack Meridian Health Network, Edison, NJ 08820, USA; 2 Hackensack Meridian School of Medicine, Hackensack Meridian Health Network, Nutley, NJ 07110, USA

**Keywords:** thymic neoplasia, thymic cancer, HITHOC, robotic-assisted surgery, thoracic surgery

## Abstract

**Background/Objectives**: Thymic epithelial tumors with pleural metastasis require a multimodal treatment approach with the use of novel modalities such as hyperthermic intrathoracic chemotherapy (HITHOC). This systematic review and case report aims to summarize the existing evidence regarding HITHOC for these tumors and presents the first case of a robotic approach to HITHOC. **Methods**: A search in November 2023 yielded a total of 17 articles, including 281 patients who met the eligibility criteria (i.e., underwent HITHOC for treatment of a thymic epithelial tumor). **Results**: Variations existed among HITHOC regimens and surgical approaches. The most common complications observed were air leaks. Overall survival ranged 92–95% at 1 year, 83–91.7% at 3 years, 66.7–92% at 5 years, 40–83.3% at 10 years, and 27.8–58.2% at 15 years. **Conclusions**: While HITHOC for thymic epithelial tumors with pleural dissemination has been shown to yield successful outcomes in the literature, this procedure has historically been performed almost exclusively via an open thoracotomy. The robotic approach to HITHOC is feasible and affords several important benefits.

## 1. Introduction

Thymic epithelial tumors (TETs) are a group of tumors that arise from epithelial cells of the thymic gland in the anterior mediastinum. Although it is a rare disease with incidence rates estimated at 0.13 and 0.31/100,000 annually, it is the most common primary neoplasm in the anterior mediastinum [1,2]. TETs include thymoma, thymic carcinoma, and neuroendocrine tumors of the thymus, all of which are histologically classified by the World Health Organization (WHO) [3]. TETs have the potential to invade adjacent organs and blood vessels, or, in more advanced stages, spread to the pleura and pericardium or disseminate through lymphatic or hematogenous routes.

TET staging at the time of diagnosis (historically clinically staged by the Masaoka–Koga system and recently replaced by the TNM/American Joint Committee on Cancer (AJCC) system) is the most important prognostic parameter [4]. Surgical resection is the first step and the mainstay of treatment for TETs, with complete surgical resection (R_0_) providing the best prognosis [2]. The tumor stage and ability to achieve R_0_ then dictates recommendations for further treatment with radiotherapy and chemotherapy. Despite best efforts, 10–40% of patients with TET experience recurrence, most commonly in the pleural space [5,6].

TETs with pleural dissemination (Masaoka–Koga Stage IV or AJCC Stage IV) are particularly difficult to treat and cure. Pleural dissemination can occur in TETs both de novo/primarily at initial diagnosis [DNT, de novo stage IV thymoma] or at relapse after definitive treatment for initial TET [TPR, thymoma with pleural recurrence].

The recalcitrant nature of TET treatment has motivated the use of novel modalities as adjuncts to surgical resection. This includes hyperthermic intrathoracic chemotherapy (HITHOC) for tumors with pleural dissemination, which acts similarly to hyperthermic intraperitoneal chemotherapy (HIPEC) for peritoneal dissemination of tumors. The efficacy of HITHOC treatment lies in its ability to deliver local doses of chemotherapy at high temperatures. In addition to direct cytotoxicity, HITHOC has been shown to activate the anti-neoplastic immune system response [7].

The first case of HITHOC for a TET was reported in 1999 [8]. Its subsequent use in the treatment of primary and recurrent TETs with pleural dissemination, both de novo and in relapse, has been highlighted in multiple studies over the last two decades. However, there has been little discourse around the evolution of conventional techniques of HITHOC delivery in the modern era of minimally invasive thoracic surgery. This review aims to summarize the existing evidence regarding HITHOC for both thymoma and thymic carcinoma and offers new insights into modern minimally invasive techniques for HITHOC, informed by the literature and our experience.

## 2. Materials and Methods

This systematic review was conducted in accordance with the Preferred Reporting Items for Systematic Reviews and Meta-Analyses (PRISMA) 2020 guidelines to ensure the highest quality of evidence synthesis.

### 2.1. Search Strategy

A literature search of MEDLINE (PubMed) was conducted in November 2023 using the search string shown in Supplementary Section S1. We included all original, full-text articles reporting on patients diagnosed with thymoma or thymic carcinoma and receiving intraoperative HITHOC as part of their management. We excluded studies with full texts in a language other than English.

### 2.2. Study Selection

**Figure 1:**
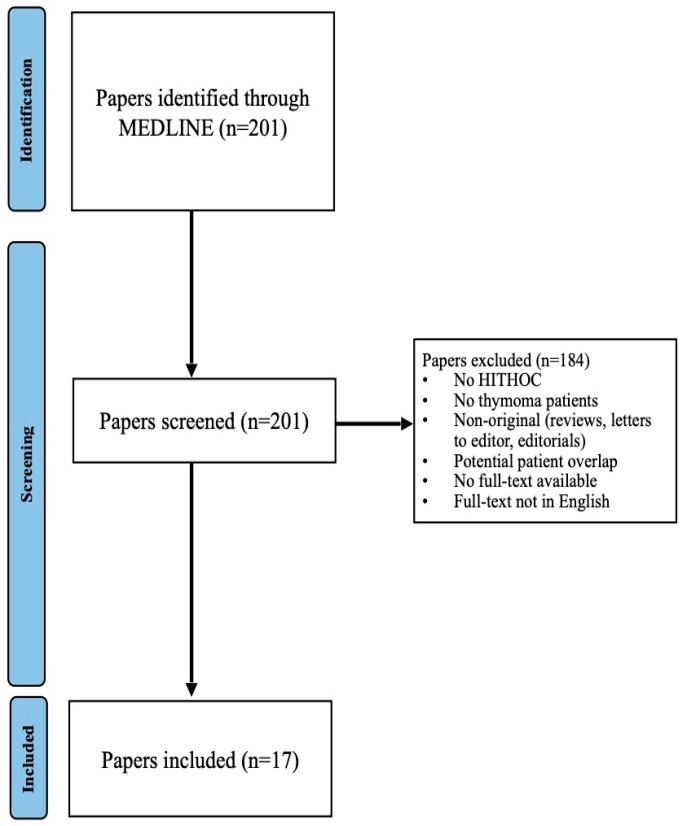
PRISMA Flowchart for study selection.

A total of 201 unique articles were identified via the search string and assessed for eligibility. Two reviewers (RSM, EC) screened all titles and abstracts independently, and ascertained their eligibility for inclusion based on the selection criteria. In case of discrepancies, a third reviewer (FB) was consulted, and a final consensus decision was reached. A total of 21 articles were initially shortlisted. However, four articles were excluded, as they likely reported on patient cohorts that had been described in other articles (i.e., publications from the same team and institutions across overlapping time periods); the most recently published articles and with larger sample size were included (i.e., to prevent double counting patients). We assumed that patients in the most recent articles were also included in the previous articles from the same study teams and institutions due to identical inclusion criteria and overlapping time periods. Ultimately, 17 papers were included for data extraction and synthesis (Figure 1).

### 2.3. Data Extraction

Data extraction was performed independently by two reviewers (RSM, EC) and recorded in a Google Drive data extraction sheet. The information extracted is shown in Supplementary Section S2. The extracted data are qualitatively presented in the form of tables.

### 2.4. Risk of Bias Assessment

Risk of bias was evaluated via the National Institutes of Health (NIH) Quality Assessment Tool for Observational Cohort and Cross-Sectional Studies. A self-chosen quantitative threshold of ≥75% was used to categorize studies as good quality, ≥50% to <75% to categorize studies as fair quality, and <50% to categorize studies as poor quality.

## 3. Results

A total of 201 articles were initially identified using our search strategy. A total of 17 articles published between 2002 and 2023 and including 281 patients [6,9,10,11,12,13,14,15,16,17,18,19,20,21,22,23,24] were shortlisted for inclusion in this systematic review (Figure 1). These included published articles from the United States of America [12,15], European countries [12,15], Japan [14], China [21], India [22,23], and Israel [18,20]. The risk of bias assessment for the included studies is presented in Supplementary Section S3. Only one study was deemed to be of good quality [19], with the remainder being of fair quality [6,9,10,11,12,13,14,15,16,17,18,20,21,22,23,24].

The study and patient characteristics are shown in Table 1, and tumor characteristics are shown in Table 2. All of the studies included patients with TETs with pleural dissemination (Masaoka Stage IV or TNM IV) who underwent surgical cytoreduction followed by HITHOC. Four studies reported exclusively on patients with pleural dissemination with TET recurrence following prior resection of the primary tumor (TPR) [6,9,10,24]. Five studies reported exclusively on patients with de novo pleural dissemination of TET found on initial diagnosis (DNT) [13,15,16,17,22]. Eight studies included both TPR and DNT patients [11,12,14,18,19,20,21,23]. The Masaoka–Koga tumor stage that is included in Table 2 references the tumor stage of the primary tumor of patients who received HITHOC for their recurrent TET with pleural involvement (TPR). Given that, at the time of HITHOC, all of the included patients had either TET with primary pleural dissemination (DNT) or pleural recurrence (TPR), they are all assumed to be Masaoka Stage IV/TNM Stage IV.

### 3.1. HITHOC Protocols and Renoprotective Strategies

Cisplatin was commonly administered as monotherapy, with doses ranging from 100 to 225 mg/m^2^ [12,15,17,19,20,21,22,23] or fixed doses of 50-200 mg [13,14,20].Cisplatin was also administered in combination with other chemotherapeutic agents, with cisplatin doses ranging from 50 to 175 mg/m^2^ [6,9,10,11,16,17,19,23,24] or 1 mg/kg [16] when in combination.

Drugs commonly used in combination with cisplatin included doxorubicin (25 mg/m^2^ or a fixed dose of 50–65 mg) [9,20,23], epirubicin (25 mg/m^2^) [6], adriamycin (15–25 mg/m^2^ or a fixed dose of 60–100 mg) [10,17], and mitomycin (25 mg/m^2^, 0.7 mg/kg [maximum dose of 60 mg], or a fixed dose of 15 mg) [11,16,17]. This information is summarized in Table 3.

HITHOC was administered intraoperatively at mean temperatures ranging from 40 to 44 °C [6,9,10,13,14,15,16,17,18,19,20,21,22,23,24], with maximum temperatures up to 48.2 °C being described [16], and for durations ranging between 60 and 120 min [6,9,10,11,12,13,14,15,16,17,18,19,20,21,22,23,24]. Circulatory flow rates for the HITHOC ranged from 200 to 2500 mL/min [10,13,15,18,19,20,21,23,24]. Although most HITHOC protocols were implemented concurrent with operative cytoreduction, Kodama et al. [14] performed HITHOC in the postoperative setting after the cytoreductive operation, while Yellin et al. reserved postoperative HITHOC for patients with intraoperative hemodynamic instability [20].

Renoprotective measures described adequate hydration [6,9,17], including forced diuresis (>50–100 mL/h) intraoperatively [15,20] and for the first five days after HITHOC [13,23]. Maintenance of mean arterial pressure above 60 mm Hg was also targeted [13]. Intravenous amifostine (910 mg/m^2^) may be given 30 min prior to HITHOC, with sodium thiosulfate directly after HITHOC (4 or 9 g/m^2^) and continuously over the next 6 h (12 g/m^2^) at the intensive care unit [11,15,23]. Some protocols included twice daily renal function tests in the postoperative period [6,9,22].

Most studies described cases in which thoracotomy (most commonly posterolateral, but also anterolateral) or sternotomy was performed for tumor resection prior to HITHOC initiation [6,9,10,11,12,13,14,15,16,17,18,19,20,22,23]. Only one study, Yu et al., reported using video-assisted thoracoscopic surgery for tumor resection [21]. In an attempt to achieve complete surgical cytoreduction, many studies also reported concurrent resection intrathoracic structures, such as the pleura (58.3–100%) [6,9,10,11,12,13,14,15,16,17,18,19,20,21,22,23], diaphragm (0–60%) [9,10,11,12,13,15,17,19,20,22], pericardium (0–100%) [10,12,13,15,19,22], lung (pneumonectomy: 6.7–33%; subtotal lung resection: 11.4–60%) [10,11,14,18,20,21,22,23], and chest wall (5.2–20%) [19,20]. The operative approach for surgical cytoreduction and HITHOC administration is outlined in Supplementary Materials.

### 3.2. Adverse Events 

No patients experienced intraoperative hemodynamic, respiratory, thermal instability, or any other signs of chemotoxicity intraoperatively. The most common complications observed were air leaks (7.6%) and bleeding (7.2%), with 6.8% of patients being taken back to the operating room. Only 3.7% of patients experienced nephrotoxicity. Other uncommon (incidence of approximately 1% or less) postoperative complications included pyothorax, deep vein thrombosis, pulmonary embolism, cytotoxicity, wound dehiscence, pleural effusion, and wound infection. The early postoperative mortality rate was 0.9%. These results are shown in Table 4.

### 3.3. Follow-Up Regimens

Patients were seen for clinical follow-up at 1–1.5 [22], 3 [9,10,16], 6 [11], and 12 [15] months after hospital discharge, and subsequently every 3–6 months until 5 years [10] or life [15,17,19,20]. Imaging protocols included chest and abdominal CT (with or without PET scans) at 3 months post-discharge [10], followed by every 3–6 months for the first few years [6,12,22], and subsequently every 6 months [16,20] or annually for 10 years [9] or life [6,11,12,22]. MRI can be performed to distinguish between pleural scar formation and recurrent disease [20].

### 3.4. Clinical and Oncologic Outcomes

The average length of intensive care unit stay ranged from 1 to 3 days [10,11,13,19,22,23,24], average duration of chest tube drainage ranged from 5 to 7.1 days [9,11,13,22], and the average length of hospital stay ranged from 7 to 18 days [6,9,10,11,12,13,15,16,18,19,22,23,24].

The average duration of follow-up ranged from 8 to 70.9 months [6,9,10,12,14,15,16,17,18,20,21,22,23,24]. For studies reporting data on DNTs, the average disease-/recurrence-free interval ranged from 49 to 72.2 months [11,13] and overall survival ranged from 100% at 1 year [11], 80.8–100% at 5 years [11,20], to 33–72.7% at 10 years [11,20]. Disease-/recurrence-/progression-free survival was 60.6% at 5 years [20] and 43.3% at 10 years [20]. Ten-year disease-specific survival was 79% [20].

For studies reporting data on TPRs, the average disease-/recurrence-free interval ranged from 53 to 88 months [6,9,11,24], and overall survival ranged 88–93% at 1 year [11,24], 66.7–92% at 5 years [9,11,20,24], and 49–77% at 10 years [6,11,20]. Disease-/recurrence-/progression-free survival ranged 46.9–91% at 5 years [6,9,20] and 17.9–37.5% at 10 years [6,20]. Ten-year disease-specific survival was 87.5% [20].

Aprile et al. [6] compared patients undergoing surgery alone versus those undergoing surgery plus HITHOC, and found comparable overall survival but longer disease-free interval in the surgery plus HITHOC group. Dolan et al. [12] and Kumar et al. [22] performed a similar comparison and found no significant differences in overall survival [12,22], disease-free survival [12], or local recurrence-free survival [12] between patients undergoing surgery alone versus surgery plus HITHOC. Chappuy et al. and Markowiak et al. compared patients with stage IVA de novo and IVA distant relapse pleural spread of thymoma undergoing HITHOC, and found comparable overall survival [11,23] but a longer disease-free interval [11] in patients undergoing HITHOC for distant pleural relapse of stage IV thymoma. Ried et al. [19] compared patients with thymic carcinoma versus thymoma undergoing HITHOC and demonstrated superior overall and recurrence-free/progression-free survival in patients with thymoma. These results are shown in Table 5. 

## 4. Discussion: Our Experience with Minimally Invasive Delivery of HITHOC

We recently performed HITHOC with robot-assisted total parietal pleurectomy, total decortication with resection of multiple visceral pleural nodules, and wedge resection of the left lower lobe for a 56-year-old patient with primary (de novo) WHO B2/B3 and Masaoka Stage IV/AJCC TNM Stage IVB thymoma. This patient had previously had a robotic resection of the primary tumor with associated mediastinal lymph node resection and excision of multiple pleural masses 6 months prior. This initial procedure was mainly intended to be diagnostic and cytoreductive. There were innumerable millimeter-sized lesions present throughout the pleura and chest wall that were not resected. The pathologic stage of the tumor was judged to be pT3NxM1b.

The current procedure aimed to eradicated residual disease in the chest through HITHOC and surgical cytoreduction via the daVinci Xi robotic surgical system. The procedure was performed with the patient in the right lateral decubitus position with the robot initially docked pointing caudad. Two 8 mm ports were placed in the left 7th and 8th intercostal spaces and a third 12 mm accessory port was placed in the left 10th intercostal space. A fenestrated bipolar, dipolar dissector, and tip-up grasper were used to complete the surgical cytoreduction. Parietal pleurectomy was performed starting with the inferior aspect of the chest including the costophrenic angles, with complete stripping of all pleura and associated parietal pleural nodules en bloc. Several adhesions to the lung were taken down to create working room within the chest. Multiple small nodules over the diaphragm were resected using cautery. Once cytoreduction and parietal pleurectomy in the inferior aspect of the chest cavity were completed, the robot was undocked and re-oriented to point cephalad to be able to work in the superior aspect of the chest cavity for the completion of the parietal pleurectomy. Once this was complete, the entire parietal pleura was removed en bloc from the chest cavity. Following this, multiple pulmonary nodules were resected from the surface of the lung. We removed each of these nodules along with the associated visceral pleura and, in some cases, with associated superficial lung parenchyma where the visceral pleura was thin or non-existent. There were dozens of such nodules on the surface of the upper and lower lung lobes. Finally, due to the presence of numerous pleural nodules on the surface of the lower edge of the lower lobe, we performed a wedge resection of this region to remove these conglomerated nodules en bloc. All resected specimens were removed in an Endo Catch^TM^ Specimen Retrieval Pouch.

**Figure 2:**
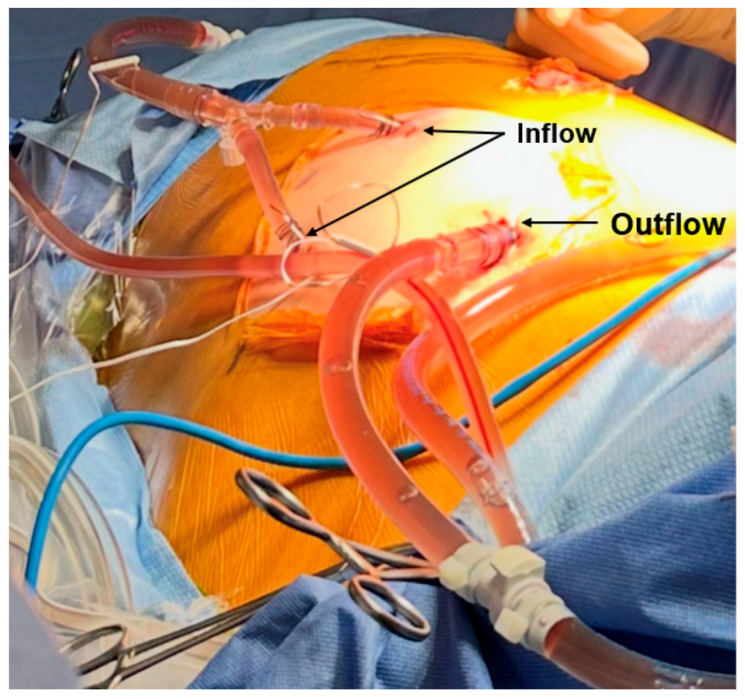
Inflow and outflow ports of the HITHOC system. The inflow ports actively pump solution into the peritoneal cavity, while the outflow port passively drains the fluid, maintaining a closed-loop, continuation circulation.

The administration of HITHOC was begun after gross resection of the tumor was completed. The inflow and outflow cannulas were placed through the lateral-most airtight robotic ports (Figure 2). We used a combination of cisplatin (80 mg/m^2^ for a total of 180 mg) and adriamycin (25 mg/m^2^ for a total of 55 mg). The inflow and outflow temperatures were 42 °C and 40.5 °C, respectively, and cooling blankets were used to keep the core temperature below 38 °C. The HITHOC was circulated for a total of 60 min. Following HITHOC, 0.75 L of heated povidone-iodine (10% concentration) was combined with 0.75 L of ringers lactate and circulated for 5 min. The use of a povidone-iodine intra-thoracic lavage has been described for use in pleural dissemination of thymoma and malignant pleural mesothelioma, but its use remains nascent and its benefit debated [24,25]. Finally, the chest cavity was irrigated with 5 L of saline. The total procedure lasted 327 min and was without intraoperative complications. Estimated total blood loss was 50 mL. The patient was extubated immediately postoperatively while on the operating table and was brought to recovery with an anterior and posterior chest tube in place.

The patient had an uneventful postoperative course at the hospital, barring a single event of supraventricular tachycardia that was treated with amiodarone. Ambulation and diet were started on postoperative day 0. The posterior chest tube was removed on postoperative day 2, followed by removal of the anterior chest tube on postoperative day 3, at which point the patient was discharged home. This length of stay is notably lower than the average range of 7–18 days reported in the literature [6,9,10,11,12,13,15,16,18,19,22,23,24] and emphasizes the quicker recovery afforded by a minimally invasive approach to HITHOC. For our patient, there was no evidence of recurrent disease at the last CT scan, which was performed at the 17-month postoperative time point.

As HITHOC procedures may be accompanied by concurrent resection of local mediastinal structures, such as the pleura, pericardium, diaphragm, lung, or chest wall, it is important to consider the options for approach towards surgical resection. Most studies in our systematic review employed an open thoracotomy or sternotomy approach, barring a single study from China that used video-assisted thoracoscopic surgery [21]. Our review did not yield any studies that described a robotic approach to HITHOC and concurrent resections. With the increasing ubiquitousness of minimally invasive thoracic surgery, particularly robotic surgery, surgeons must consider these approaches instead of conventional open operations for procedures that require significant resection of local mediastinal structures.

In addition to the well-known benefits of minimally invasive surgery (i.e., safer operations, less pain, faster recovery, and earlier discharge), the robotic approach to HITHOC affords several important benefits compared to conventional open thoracotomy. The use of robotic ports for the inflow and outflow of chemotherapy provides a closed-loop circuit that minimizes the escape of chemotherapy aerosol or spillage of chemotherapy in the operating room, thus reducing the risk of exposure for the surgical team. The use of the robot allows for magnified visualization and superior operative access within the thoracic cavity than possible with conventional video-assisted thoracoscopic surgery. These advantages may allow for a more successful cancer operation with R_0_ resections, although future studies are needed for more definitive conclusions. While case-to-case variability is expected, the robotic approach is likely possible and feasible for most TETs with pleural dissemination. It may be less feasible, however, in very obese patients (due to limitations in instrument maneuverability and mobility due to increased chest wall thickness) and in very small or young patients (due to limited space for instrumentation).

### Limitations

This systematic review is limited by the heterogeneity of the included studies, which stemmed from the small sample sizes included in the individual studies. Moreover, the differing chemotherapy regimens administered during HITHOC across the various institutions and studies included in our review also leave room for ambiguity with regards to the most optimal HITHOC protocol. In addition, only one study was deemed to be of good quality, with the remainder being of fair quality; this impacts the strength of the conclusions that may be drawn from the synthesized evidence and emphasizes the need for robust research in the future. The review included patients with both primary and recurrent thymic epithelial tumors with pleural dissemination, and the field would benefit from additional studies looking at each group separately, as the outcomes may differ. It is also important to note that, given the relatively indolent nature of TETs, it is possible that overall survival outcomes do not completely reflect the disease course, and in studies that only reported on overall survival, recurrences may have occurred. However, few studies reported time-to-recurrence or progression-free survival as outcomes. In addition, we were not able to perform a pooled analysis/meta-analysis for most of the categorical outcomes. This limits our ability to draw definitive conclusions regarding the efficacy of HITHOC, especially as studies such as Aprile, Dolan, and Kumar showed comparable results with regards to the efficacy of HITHOC vs. surgical cytoreduction alone [6,12,22]. Lastly, we restricted our search to MEDLINE (PubMed)-indexed articles only, which may have led to the exclusion of more obscure literature published in non-PubMed-indexed journals.

## 5. Conclusions

Our qualitative synthesis of the existing literature demonstrated somewhat mixed results for the use of HITHOC accompanied by surgical cytoreduction in the management of TETs with pleural dissemination. However, the lack of adequately powered comparative studies limits our ability to make definitive conclusions regarding the effectiveness of this strategy as opposed to surgical cytoreduction alone. The vast majority of studies reported the use of conventional open techniques of HITHOC delivery, which is accompanied by long hospital stays and frequent complications such as air leaks, bleeding, and early reoperations. We described our experience with minimally invasive delivery of HITHOC via robotic ports, which is the first reported case of its kind to the best of our knowledge. Given several key advantages of such an approach, which include patient safety, superior clinical outcomes, and safety of the surgical team, we believe that the minimally invasive delivery of HITHOC could represent the next paradigm of surgical care for TETs with intrathoracic dissemination.

## Figures and Tables

**Table 1:** General study and study participant characteristics. * Percentage of male patients is described for the overall study cohort undergoing HITHOC, not the TET subset. *MG*: Myasthenia gravis; *PRCA*: pure red cell aplasia.

First Author (Year Published)	Enrollment Period	County of Study	Sample Size	Sex (% Male)	Inclusion Criteria	Paraneoplastic Syndromes (%)
Ambrogi et al. (2016) [9]	2005–2012	Italy	13	7/13 (53.4%)	Patients with a history of TET s/p prior resection of primary tumor with pleural recurrence of TET who underwent surgical cytoreduction followed by HITHOC	MG: 8/13 (61.5%) PRCA: 1/13 (7.7%)
Aprile et al. (2020) [6]	2005–2017	Italy	27	9/27 (33.3%)	Patients with a history of TET s/p prior resection of primary tumor with pleural recurrence of TET who underwent surgical cytoreduction followed by HITHOC	MG: 23/27 (85.2%)
de Bree et al. (2022) [10]	1998–2000	The Netherlands	3	0/3 (0%)	Patients with a history of TET s/p prior resection of primary tumor with pleural recurrence of TET who underwent surgical cytoreduction followed by HITHOC	MG: 1/3 (33.3%)
Chappuy et al. (2022) [11]	1997–2021	France	40	15/40 (37.5%)	Patients with pleural involvement of TET (both de novo and pleural recurrence following primary resection) who underwent surgical cytoreduction followed by HITHOC	MG: 17/40 (42%)
Dolan et al. (2022) [12]	1990–2020	USA	12	3/12 (25%)	Patients with pleural involvement of TET (both de novo and pleural recurrence following primary resection) who underwent surgical cytoreduction followed by HITHOC	-
Klotz et al. (2021) [13]	2014–2018	Germany	12	63/76 * (82.9%)	Patients with TET with de novo pleural involvement who underwent surgical cytoreduction followed by HITHOC	-
Kodama et al. (2013) [14]	1987–2010	Japan	12	7/12 (58.3%)	Patients with pleural involvement of TET (both de novo and pleural recurrence following primary resection) who underwent surgical cytoreduction followed by HITHOC	MG: 0/12 (0%)
Kumar et al. (2021) [22]	2015–2018	India	6	5/6 (83.3%)	Patients with TET with de novo pleural involvement who underwent surgical cytoreduction followed by HITHOC	MG: 4/6 (66.7%)
Markowiak et al. (2021) [23]	2008–2017	Germany	29	17/29 (58.6%)	Patients with pleural involvement of TET (both de novo and pleural recurrence following primary resection) who underwent surgical cytoreduction followed by HITHOC	-
Maury et al. (2017) [24]	1997–2015	France	19	8/19 (42.1%)	Patients with a history of thymoma (thymic carcinoma excluded) s/p prior resection of primary tumor with pleural recurrence of thymoma who underwent surgical cytoreduction followed by HITHOC	MG: 9/19 (47.4%)
Miller et al. (2023) [15]	2014–2021	USA	14	14/35 * (40%)	Patients with TET with de novo pleural involvement who underwent surgical cytoreduction followed by HITHOC. Additional inclusion criteria: Primary disease site under control for at least 12 months Tumor pleural thickness less than 2 cm Non-fissural pleural disease No mediastinal lymph node involvement No chest wall invasion	-
Monneuse et al. (2003) [16]	1990–2000	France	1	0/1 (0%)	Patients with TET with de novo pleural involvement who underwent surgical cytoreduction followed by HITHOC	-
Patel et al. (2018) [17]	2011–2018	India	1	1/1 (100%)	Patients with TET with de novo pleural involvement who underwent surgical cytoreduction followed by HITHOC. Additional inclusion criteria: No bulky mediastinal involvement No extra-thoracic extension of disease No massive infiltration of the lung, or No sarcomatoid histology	-
Refaely et al. (2011) [18]	1995–2000	Israel	15	11/15 (73.3%)	Patients with pleural involvement of TET (both de novo and pleural recurrence following primary resection) who underwent surgical cytoreduction followed by HITHOC	MG: 5/15 (33.3%)
Ried et al. (2023) [19]	2008–2019	Germany	58	36/58 (62.1%)	Patients with pleural involvement of TET (both de novo and pleural recurrence following primary resection) who underwent surgical cytoreduction followed by HITHOC	-
Yellin et al. (2013) [20]	1995–2012	Israel	35	25/35 (71.4%)	Patients with pleural involvement of TET (both de novo and pleural recurrence following primary resection) who underwent surgical cytoreduction followed by HITHOC	MG: 15/35 (42.9%)
Yu et al. (2013) [21]	2008–2012	China	4	1/4 (25%)	Patients with pleural involvement of TET (both de novo and pleural recurrence following primary resection) who underwent surgical cytoreduction followed by HITHOC	MG: 1/4 (25%)

**Table 2:** Tumor and treatment characteristics. TPR: Thymoma Pleural Relapse. † For studies that looked at TPR and included stage of primary tumor prior to HITHOC treatment of Masaoka–Koga/TNM stage IVA recurrence. * Percentage of patients is described for the overall study cohort undergoing HITHOC, not the TET subset.

Study	Additional Treatment Details	WHO Histologic Classification	Side of Metastasis	Masaoka–Koga Primary Tumor Stage ^†^	Local Infiltration
Ambrogi et al. (2016) [9]	Average between primary thymectomy and pleural recurrence of thymic malignancy: 47.2 months Two patients had previous TPR surgically treated after 36 and 104 months, and then ipsilateral new recurrences were treated with HITHOC after 203 and 39 months Radiation prior to surgical resection of TPR and HITHOC: 2/13 (15.4%)	A: 1/13 (7.7%) AB: 2/13 (15.4%) B1: 2/13 (15.4%) B2: 3/13 (23.1%) B3: 4/13 (30.8%) B2/B3: 1/13 (7.7%)	-	IIA: 2/13 (15.4%) IIB: 5/13 (38.5%) III: 5/13 (38.5%) IVA: 1/13 (7.7%)	Diaphragm: 6/13 (46.2%)
Aprile et al. (2020) [6]	Adjuvant therapy after primary surgery: Radiotherapy: 18/27 (66.7%) Chemotherapy: 3/27 (11.1%) Neoadjuvant therapy before primary surgery: Radiotherapy: 1/27 (3.7%) Chemotherapy: 3/27 (11.1%) Radicality of primary surgery: R0: 21/27 (77.8%) R+: 6/27 (22.2%) Adjuvant therapy after surgery for TPR: Radiotherapy: 4/27 (14.8%) Chemotherapy: 2/27 (7.4%) Neoadjuvant chemotherapy before surgery for TPR: 1/27 (3.7%)	A: 2/27 (7.4%) AB: 2/27 (7.4%) B1: 4/27 (14.8%) B2: 8/27 (29.6%) B2/B3: 4/27 (14.8%) B3/B2: 2/27 (7.4%) B3: 5/27 (18.5%)	-	IIA: 2/27 (7.4%) IIB: 8/27 (29.6%) III: 11/27 (407%) IVA: 6/27 (22.2%)	Visceral Pleura: 17/27 (63%) Parietal Pleural: 25/27 (92.6%) Pericardium: 8/27 (29.6%) Lung Parenchyma: 4/27 (14.8%) Diaphragm: 17/27 (63%)
de Bree et al. (2022) [10]	Adjuvant chemotherapy after primary surgery: 1/3 (33.3%) Adjuvant radiotherapy after primary surgery: 1/3 (33.3%)	-	Right: 1/3 (33.3%) Left: 1/3 (33.3%) Bilateral: 1/3 (33.3%)	-	-
Chappuy et al. (2022) [11]	-	A: 2/40 (5%) AB: 1/40 (2.5%) B1: 3/40 (7.5%) B1/B2: 2/40 (5%) B2: 18/40 (45%) B2/B3: 5/40 (12.5%) B3: 9/40 (22.5%)	-	-	-
Dolan et al. (2022) [12]	Neoadjuvant therapy: Chemotherapy: 9/12 (75%) Chemoradiation: 1/12 (8.3%) Adjuvant therapy: Chemotherapy: 1/12 (8.3%) Radiation: 3/12 (25%)	A: 1/12 (8.3%) AB: 2/12 (16.7%) B2: 2/12 (16.7%) B2B3: 2/12 (16.7%) B3: 2/12 (16.7%)	-	-	-
Klotz et al. (2021) [13]	-	B1: 1/12 (8.3%) B2: 6/12 (50%) B3: 2/12 (16.7%) Thymic Carcinoma: 3/12 (25%)	Left: 34.2% * Right: 65.8% *	-	-
Kodama et al. (2013) [14]	-	-	-	-	-
Kumar et al. (2021) [22]	Neoadjuvant chemotherapy: 6/6 (100%) Adjuvant radiotherapy: 6/6 (100%)	-	Right: 2/6 (33.3%) Left: 4/6 (66.7%)	-	-
Markowiak et al. (2021) [23]	Neoadjuvant “induction” therapy: 15/29 (51.7%)	B1: 4/29 (13.8%) B2: 10/29 (34.5%) B2/B3: 5/29 (17.2%) B3: 7/29 (24.2%) C: 3/29 (10.3%)	Right: 15/29 (51.7%)	-	-
Maury et al. (2017) [24]	Neoadjuvant chemotherapy prior to initial thymectomy: 5/19 (26.3%) Adjuvant radiotherapy after initial thymectomy: 8/19 (42.1%)	B1: 2/19 (10.5%) B2: 10/19 (52.6%) B3: 5/19 (26.3%) B2/B3: 2/19 (10.5%)	-	Stage I/II: 1/19 (5.3%) Stage III: 9/19 (47.4%) Stage IV: 9/19 (47.4%)	-
Miller et al. (2023) [15]	-	-	-	-	-
Monneuse et al. (2003) [16]	No adjuvant radiotherapy	-	-	-	-
Patel et al. (2018) [17]	Adjuvant chemotherapy: 1/1 (100%)	-	-	-	-
Refaely et al. (2011) [18]	Neoadjuvant chemotherapy: 8/15 (53.3%) Previous surgical resection of thymoma: 7/15 (46.7%)	Mixed Thymoma: 5/15 (33.3%) Lymphocytic Thymoma: 3/15 (20%) Epithelial Thymoma: 2/15 (13.3%) Thymic Carcinoma: 4/15 (26.7%) Carcinoma in Thymic Cyst: 1/15 (6.7%)	Unilateral: 14/15 (93.3%) Bilateral: 1/15 (6.7%)		-
Ried et al. (2023) [19]	Neoadjuvant chemotherapy: 20/58 (34.5%) Adjuvant chemotherapy: 7/58 (12.1%) Additive chemotherapy: 25/58 (43.1%) Adjuvant radiotherapy: 13/58 (22.4%)	AB: 1/58 (1.7%) B1: 8/58 (13.8%) B2: 24/58 (41.4%) B3: 9/58 (15.5%) Thymic Carcinoma: 15/58 (25.9%) Atypical carcinoid of the thymus: 1/58 (1.7%)	Left: 29/58 (50%) Right: 29/58 (50%)	-	-
Yellin et al. (2013) [20]	Neoadjuvant chemotherapy: 17/35 (48.6%) Adjuvant chemotherapy: 7/35 (20%) Adjuvant radiotherapy: 10/35 (28.6%)	AB: 2/35 (5.7%) B1: 5/35 (14.3%) B2: 17/35 (48.6%) B3: 7/35 (20%) Thymic Carcinoma: 4/35 (11.4%)	Right: 14/35 (40%) Left: 20/35 (57.1%) Bilateral: 1/35 (2.9%)	-	-
Yu et al. (2013) [21]	Adjuvant radiation: 4/4 (100%)	-	Right: 2/4 (50%) Left: 2/4 (50%)	-	-

**Table 3:** HITHOC protocol. ^1^ One patient with shortened duration of 30 min. IQR: Interquartile range.

Study	Chemotherapeutic Agent(s)	Dose (mg/m^2^ Unless Otherwise Specified)	Duration (Minutes)	Perfusion Rate
Ambrogi et al. (2016) [9]	Cisplatin in combination with Doxorubicin	Cisplatin: 80 Doxorubicin: 25	60	-
Aprile et al. (2020) [6]	Cisplatin in combination with Epirubicin	Cisplatin: 80 Epirubicin: 25	60	**-**
de Bree et al. (2022) [10]	Cisplatin in combination with Doxorubicin	Cisplatin: 50–80 Doxorubicin: 15–25	90	1000 mL/min
Chappuy et al. (2022) [11]	Cisplatin in combination with Mitomycin	Cisplatin: 50 Mitomycin 25	90	-
Dolan et al. (2022) [12]	Cisplatin alone	175	60 ^1^	-
Klotz et al. (2021) [13]	Cisplatin alone	200 mg/L	60	1000 mL/min
Kodama et al. (2013) [14]	Cisplatin alone or Carboplatin alone	Cisplatin: 50–100 mg/chest cavity Carboplatin: 450 mg/chest cavity	60	-
Kumar et al. (2021) [22]	Cisplatin alone	130–150	60	-
Markowiak et al. (2021) [23]	Cisplatin alone or Cisplatin in combination with Doxorubicin	Cisplatin alone: 100 or 150–175 Cisplatin when in combination with Doxorubicin: 175 Doxorubicin: 65 mg	60	1500 mL/min
Maury et al. (2017) [24]	Mitomycin in combination with Cisplatin	Mitomycin: 25 (maximum dose of 60 mg) Cisplatin: 50 (maximum dose of 100 mg)	90	200 mL/min
Miller et al. (2023) [15]	Cisplatin alone	225	60	1500–1700 mL/min
Monneuse et al. (2003) [16]	Mitomycin in combination with Cisplatin	Mitomycin: 0.7 mg/kg Cisplatin: 1 mg/kg	60	-
Patel et al. (2018) [17]	Cisplatin alone or Cisplatin in combination with Adriamycin or Cisplatin in combination with Mitomycin C	Cisplatin: 100–150 Adriamycin: 60–100 Mitomycin C: 15	60–90	-
Refaely et al. (2011) [18]	Cisplatin alone	100: 1/15 (6.7%) 150: 11/15 (73.3%) 180: 1/15 (6.7%) 200: 2/15 (13.3%)	60	1000–2000 mL/min
Ried et al. (2023) [19]	Cisplatin alone or Cisplatin in combination with Doxorubicin	Cisplatin: Median = 120 (IQR: 100–175) Doxorubicin: Median = 35.3 (IQR: 32.3–38.1)	60	1200 mL/min
Yellin et al. (2013) [20]	Cisplatin alone or Cisplatin in combination with Doxorubicin	Cisplatin: 100 Doxorubicin: 50–60	60 ^1^	1000–2500 mL/min
Yu et al. (2013) [21]	Cisplatin alone	100	120	1800–2300 mL/min

**Table 4:** Incidence of postoperative complications in patients undergoing HITHOC for TET.

Study	Air Leak n/N (%)	Bleeding n/N (%)	Return to Operating Room n/N (%)	Pneumonia n/N (%)	Nephrotoxicity n/N (%)	Arrhythmia n/N (%)	Myasthenic Flare n/N (%)	Early Postoperative Mortality n/N (%)
Ambrogi et al. (2016) [9]	1/13 (7.7%)	3/13 (23%)	0/13 (0%)	0/13 (0%)	0/13 (0%)	0/13 (0%)	0/13 (0%)	0/13 (0%)
Aprile et al. (2020) [6]	3/27 (11%)	7/27 (26%)	0/27 (0%)	0/27 (0%)	0/27 (0%)	0/27 (0%)	0/27 (0%)	0/27 (0%)
de Bree et al. (2022) [10]	0/3 (0%)	0/3 (0%)	0/3 (0%)	0/3 (0%)	1/3 (33%)	0/3 (0%)	0/3 (0%)	0/3 (0%)
Chappuy et al. (2022) [11]	2/40 (5.0%)	0/40 (0%)	0/40 (0%)	6/40 (15%)	4/40	0/40 (0%)	0/40 (0%)	1/40 (2.5%)
Dolan et al. (2022) [12]	7/12 (58%)	4/12 (33%)	3/12 (25%)	0/12 (0%)	1/12 (8.3%)	3/12 (25%)	0/12 (0%)	0/12 (0%)
Klotz et al. (2021) [13]	-	-	-	-	0/12 (0%)	-	-	0/12 (0%)
Kodama et al. (2013) [14]	-	-	-	-	0/12 (0%)	-	-	0/12 (0%)
Kumar et al. (2021) [22]	1/6 (17%)	0/6 (0%)	0/6 (0%)	0/6 (0%)	1/6 (17%)	2/6 (33%)	1/6 (17%)	0/6 (0%)
Markowiak et al. (2021) [23]	0/29 (0%)	1/29 (3.5%)	6/29 (21%)	0/29 (0%)	2/29 (6.9%)	0/29 (0%)	0/29 (0%)	1/29 (3.5%)
Maury et al. (2017) [24]	0/19 (0%)	0/19 (0%)	0/19 (0%)	1/19 (5.3%)	2/19 (11%)	0/19 (0%)	0/19 (0%)	0/19 (0%)
Miller et al. (2023) [15]	-	-	-	-	0/14 (0%)	-	-	0/14 (0%)
Monneuse et al. (2003) [16]	0/1 (0%)	0/1 (0%)	0/1 (0%)	0/1 (0%)	0/1 (0%)	0/1 (0%)	0/1 (0%)	0/1 (0%)
Patel et al. (2018) [17]	0/1 (0%)	0/1 (0%)	0/1 (0%)	0/1 (0%)	0/1 (0%)	0/1 (0%)	0/1 (0%)	0/1 (0%)
Refaely et al. (2011) [18]	1/15 (6.7%)	1/15 (6.7%)	1/15 (6.7%)	0/15 (0%)	0/15 (0%)	0/15 (0%)	1/15 (6.7%)	0/15 (0%)
Ried et al. (2023) [19]	3/58 (5.2%)	2/58 (3.5%)	8/58 (14%)	8/58 (14%)	4/58 (6.9%)	2/58 (3.5%)	0/58 (0%)	1/58 (1.7%)
Yellin et al. (2013) [20]	2/35 (5.7%)	1/35 (1.9%)	0/35 (0%)	1/35 (2.9%)	0/35 (0%)	0/35 (0%)	2/35 (5.7%)	0/35 (0%)
Yu et al. (2013) [21]	0/4 (0%)	0/4 (0%)	0/4 (0%)	1/4 (25%)	0/4 (0%)	0/4 (0%)	0/4 (0%)	0/4 (0%)
All Studies	20/263 (7.6%)	19/263 (7.2%)	18/263 (6.8%)	17/263 (6.5%)	11/301 (3.7%)	7/263 (2.7%)	4/263 (1.5%)	3/301 (1.0%)

**Table 5:** Early postoperative and long-term oncologic outcomes. * Described for the overall study cohort undergoing HITHOC, not the thymic malignancy subset.

Study	Early/In-Hospital Outcomes	Long-Term Oncologic Outcomes
Ambrogi et al. (2016) [9]	*Mean hospital length of stay*: 8.4 days (standard deviation: 2.6 days) *Mean chest tube duration*: 7.1 days (standard deviation: 2.4 days)	*Mean follow-up period*: 64.6 months (median: 78, range: 13–107 months) *Pleural Relapse*: 4/13 (30.8%) patients (2/13 or 15.4% on ipsilateral side and 2/13 or 15.4% on contralateral side) on average after 45 months (median: 46, range: 20–68) of HITHOC and were successfully treated with radiation therapy. *Lymph Node Metastasis*: 1/13 (7.4%) patient developed mediastinal and celiac lymph node metastases and was treated with radiation therapy but was not disease free at most recent follow-up. *At the most recent follow-up*: ▪ *Alive*: 11/13 (85%) ▪ *Dead*: 1/13 (7.4%) patient with residual disease (R2 resection) died due to toxicity of systemic adjuvant chemotherapy 7 months after HITHOC; 1/13 (7.4%) patient died disease-free due to respiratory failure following pneumonia. ▪ *Disease*-*free* (amongst those alive): 10/11 (90.9%) *Mean overall survival*: 58 months (median: 64, range: 7–107); 5-year actuarial survival of 92%. *Median local disease-free survival*: 64 months (range: 13–107); 5-year actuarial disease-free survival of 91%.
Aprile et al. (2020) [6]	*Mean hospital length of stay*: 8.3 days (standard deviation: 5.3 days) No patients required intensive care unit admissions	*Mean follow-up period*: 70.9 ± 45.8 months. *Relapse*: 12/27 (44.4%) patients after a mean of 48.2 months (standard deviation: 44.13 months)—10/12 (83.3%) of patients with recurrences developed them locally after a mean time of 39.2 months (standard deviation: 23.2 months). ▪ Relapses were treated with redo surgery if relapses were considered technically resectable, with patients deemed unresectable receiving only chemotherapy and/or radiotherapy. *At the most recent follow-up*: ▪ *Alive*: 25/27 (92.6%) ▪ *Dead*: 2/27 (7.4%)—1/27 (3.7%) died because of thymoma and 1/27 (3.7%) died because of other causes. ▪ *Disease-free* (among those alive): 14/25 (56%) *Mean overall survival*: 153.1 months (95% confidence interval: 136.6–169.5 months); 10-year actuarial survival of 77% (standard error: 0.17%) *Mean local disease-free survival*: 88.0 ± 14.9 months. Five- and 10-year actuarial disease-free survivals were 46.9% (standard error: 0.11) and 37.5% (standard error: 0.12), respectively.
de Bree et al. (2022) [10]	*Average length of intensive care unit stay*: 3 days *Mean hospital length of stay*: 14.25 days (standard deviation: 2.5 days)	*Mean follow-up period: 18 months (median: 18 months; range: 5–31 months)* At the most recent follow-up: 3/3 (100%) patients alive without disease.
Chappuy et al. (2022) [11]	*Median length of intensive care unit stay*: 1 day (range: 1–26 days). *Median hospital length of stay*: 10 days (range: 6–36 days). *Median chest tube duration*: 5 days (range: 4–32 days)	*Median disease-free interval*: 49 months (DNT), 85 months (TPR), 70 months (DNT + TPR) *Median overall survival interval*: 94 months (DNT), 118 months (TPR), 118 months (DNT + TPR); 1-year rates were 100% (DNT), 88% (TPR), 92% (DNT + TPR); 5-year rates were 100% (DNT), 80% (TPR), 86% (DNT + TPR); 10-year rates were 33% (DNT), 49% (TPR), 40% (DNT + TPR) *Relapse*: Ipsilateral pleura (10/40; 25%), contralateral pleura (2/40; 5%), mediastinum (2/40; 5%), liver (1/40; 2.5%), axillary lymph node (1/40; 2.5%), lung (1/40; 2.5%), multi-site relapse (pericardium, lung, and spine; 1/40; 2.5%). *At most recent follow-up*: ▪ *Alive*: 25/40 (62.5%) ▪ *Dead*: 15/40 (37.5%); 4/15 (26.7%) due to septic shock induced by pneumonia, 4/15 (26.7%) due to disease progression, 2/15 (13.3%) from acute myasthenic gravis crisis, 2/15 (13.3%) due to chemotherapy-induced heart failure, 1/15 (6.6%) due to paraneoplastic syndrome, 1/15 (6.6%) due to coronavirus-disease-2019-induced acute respiratory distress syndrome, and 1/15 (6.6%) due to unknown causes.
Dolan et al. (2022) [12]	*Median hospital length of stay*: 11.5 days (interquartile range: 8.8–15.3 days) *Unexpected intensive care unit admission*: 1/12 (8.3%)	*Median follow-up interval*: 3.2 years (interquartile range: 1–6.4 years) *Time to recurrence*: Mean and median: 42.9 months (standard deviation: 35.1 months; interquartile range: 18.1–67.7 months). *5-year survivals*: Overall survival: 75.8%; disease-free survival: 76.2%; local recurrence-free survival: 85.7%.
Klotz et al. (2021) [13]	*Median length of intensive care unit stay*: 2 days (interquartile range: 2–3 days) * *Median length of chest tube drainage*: 7 days (interquartile range: 6–9 days) *Median hospital length of stay*: 16 days (interquartile range: 14–19 days) *	*Progression-free survival*: 72.2 months
Kodama et al. (2013) [14]	-	Median follow-up duration: 68 months Local Relapse: 5/12 (41.7%) Alive at the most recent follow-up: 10/12 (83.3%) Overall survival: 3-year: 11/12 (91.7%); 5-year: 11/12 (91.7%); 10-year: 10/12 (83.3%). Local recurrence-free survival: 3-year: 74.1%; 5-year 64.8%; 10-year: 54.0%
Kumar et al. (2021) [22]	*Mean length of intensive care unit stay*: 1.15 days (standard deviation: 0.9 days) *Mean length of chest tube drainage*: 6.4 days (standard deviation: 2.1 days) *Mean hospital length of stay*: 7.1 (standard deviation: 4.9 days)	*Median follow-up duration*: 49.5 months No mortality recorded (0/6; 0%).
Markowiak et al. (2021) [23]	*Median length of intensive care unit stay*: 1 day (interquartile range: 2 days) *Median hospital length of stay*: 18 days (interquartile range: 18 days)	*Mean follow-up duration*: 35.6 months (range: 0–106 months). *5-year overall survival*: 81.6% (DNT), 77.1% (TPR), 80.1% (DNT + TPR) *Median recurrence-free interval*: 69 months (95% confidence interval: 40.4–97.6; range: 0–69 months). *5-year recurrence-free survival for patients with R0/R1 resection*: 53.8% *Alive at the most recent follow-up*: 62.5%
Maury et al. (2017) [24]	*Median length of hospital stay*: 10 days (range: 7–36 days) *Median length of ICU stay*: 1 day (range: 1–26 days)	*Median follow-up period*: 39 months (range: 10–127 months) *At most recent follow-up*: ▪ *Alive*: 14/19 (73.7%) ▪ *Dead*: 5/19 (26.3%) ▪ *Alive* with no evidence of disease: 12/19 (63.2%) *Median disease-free survival of those with tumor recurrence*: 53 months *Overall survival*: Median: 63 months; 1-year: 93%; 5-year: 86% *Relapses*: 6/19 (31.6%) ▪ *Ipsilateral lymph node relapse*: 2/19 (10.5%) ▪ *Pericardial relapse*: 1/19 (5.3%) ▪ *Ipsilateral pleural metastasis*: 2/19 (10.5%) ▪ *Contralateral pleural relapse*: 1/19 (5.3%)
Miller et al. (2023) [15]	*Median hospital length of stay*: 7 days (4–31 days)	*Median follow-up duration*: 24 months (range: 4–60). *Pleural relapse*: Ipsilateral: 2/14; Contralateral: 1/14 ▪ Relapse was managed with repeat limited pleurectomy and decortication and HITHOC.
Monneuse et al. (2003) [16]	*Mean hospital length of stay*: 13 days (range: 9–29 days)	*Follow-up duration*: 56 months Alive without relapse at last follow-up
Patel et al. (2018) [17]	*No mechanical ventilation required*: 1/1 (100%)	*Follow-up duration*: 8 months Alive without relapse at last follow-up
Refaely et al. (2011) [18]	*Median hospital length of stay*: 7 days (range: 5–14 days)	*Mean follow-up duration*: 33.8 months (median: 34 months; standard deviation: 24.5 months; range: 0–70 months) *At most recent follow-up*: ▪ *Alive*: 11/15 (73.3%)—1/15 (6.7%) patient suspected to have contralateral local relapse; remainder were disease-free. ▪ *Dead*: 4/15 (26.7%)—3/4 (75%) due to disease progression; 1/4 (25%) due to leukemia.
Ried et al. (2023) [19]	*Prolonged ventilation > 24 h*: 2/58 (3.4%) Median duration of ICU stay: 2 days (interquartile range: 1–3 days) Median duration of hospital stay: 15 days (interquartile range: 12–23 days)	*Median follow-up*: 59 months *Tumor recurrence in R0/R1 resections*: 24/49 (48.9%) *Tumor progression in R2 resections*: 7/9 (77.8%) *Location of recurrence/progression*: Ipsilateral: 29/31 (93.5%); Contralateral: 2/31 (6.5%) ▪ *Loco-regional*: 24/31 (77.4%) ▪ *Distant metastasis*: 6/31 (19.4%) ▪ *Loco-regional and distant metastasis*: 1/31 (3.2%) *Therapy of recurrence/progression*: ▪ *No/unknown therapy*: 5/31 (16.1%) ▪ *Surgery*: 4/31 (12.9%) ▪ *Chemotherapy*: 13/31 (41.9%) ▪ *Radiotherapy*: 7/31 (22.6%) ▪ *Best supportive care*: 1/31 (3.2%) ▪ *Other therapy*: 2/31 *Alive at the end of most recent follow-up*: 45/58 (77.6%) *Overall survival rates*: 1-year: 95%, 3-year: 83%; 5-year: 77%. *Recurrence-free/progression-free survival*: 1-year: 89%; 3-year: 54%; 5-year: 44%. *Median disease-free interval*: 43 months (95% confidence interval: 20–66 months).
Yellin et al. (2013) [20]	-	*Thymic Carcinoma*: 4/4 (100%) patients died within 4 years due to distant disease spread (mean survival: 33 months) *Thymoma*: *Deaths*: Occurred from 7 months–14 years after HITHOC. ▪ *Deaths due to systemic disease progression*: 4 deaths at 7, 30, 51, and 106 months after surgery. ▪ One death due to a second primary neoplasm. ▪ Two deaths in myasthenic patients due to pneumonia. ▪ Two deaths attributable to thymus-related syndromes. ▪ One death due to right heart failure in elderly patient. *At last follow-up*: Median follow-up was 62 months (range: 3.5–202 months) ▪ One patient is still alive with widespread metastases. ▪ Two patients have probably been cured of early-stage malignant disease (lung and prostate). ▪ None of the 14 myasthenic patients had complete neurologic remission. *Survival*: ▪ *DNT*: Overall survival 5-year: 80.8%; 10-year: 72.7%; 15-year: 58.2%. o *Median overall survival*: 184 months (95% confidence interval: 120.5–247.5 months) o *Progression-free survival*: 5-year: 60.6%; 10-year: 43.3%. o *10-year disease-specific survival*: 79% ▪ *TPR*: Overall survival 5-year: 66.7%; 10-year: 55.6%; 15-year: 27.8%. o *Median overall survival*: 140 months (95% confidence interval: 34.3–245.7 months) o *Progression-free survival*: 5-year: 47.6%; 10-year: 17.9%. o *10-year disease-specific survival*: 87.5%
Yu et al. (2013) [21]	-	*Mean follow-up duration*: 2.25 years (standard deviation: 1.32 years) *At most recent follow-up*: ▪ *Alive with no evidence of recurrent disease*: 3/4 (75%) ▪ *Died*: 1/4 (25%)—due to heart failure (no evidence of recurrent or metastatic disease)

## Data Availability

There are no data associated with this study beyond what is published in the main text and Supplementary Materials. There are no template data collection forms associated with this study, as data were extracted directly into a spreadsheet software. There was no analytic code written specifically for the analysis performed in this review.

## Supplementary Materials

The following supporting information can be downloaded at: https://www.mdpi.com/article/10.3390/jcm14124094/s1, Section S1: Search string. Section S2: Variables extracted from shortlisted articles. Section S3: Risk of bias of included studies.

## Abbreviations

The following abbreviations are used in this manuscript:

HITHOC	Hyperthermic intrathoracic chemotherapy
HIPEC	Hyperthermic intraperitoneal chemotherapy
TPR	Thymoma with pleural recurrence
DNT	De novo thymoma with pleural dissemination
