# Supplementary material for: A Systematic Review of Heated Intrathoracic Chemotherapy for Thymic Epithelial Tumors and the First Case Report of a Robotic Approach: Could a Minimally Invasive Approach Offer a New Paradigm of Care?
Source: J Clin Med. 2025 Jun 10;14(12):4094. doi: 10.3390/jcm14124094 (PMC12194147; doi:10.3390/jcm14124094)
Supplement: Supplementary file 1 [file jcm-14-04094-s001.zip › jcm-3670135-supplementary.pdf]

A Systematic Review of Heated Intrathoracic Chemotherapy for Thymic Epithelial Tumors and the First Case Report of a Robotic Approach:

Could a Minimally Invasive Approach Offer a New Paradigm of Care?

Supplementary Material

Supplementary Section S1: Search string

(HITHOC OR intraoperative chemotherapy OR intrathoracic chemohyperthermia OR intrathoracic heated chemotherapy OR heated chemotherapy OR hyperthermic intrathoracic chemotherapy OR Isolation Perfusion Cancer Chemotherapy OR ITCH OR intrapleural perfusion hyperthermochemotherapy OR HITOC OR intraoperative hyperthermic pleural irrigation) AND (Thymoma OR thym* cancer OR thym* tumor* OR thym* malignancies OR thym* carcinoma OR thym* neoplasia)

Supplementary Section S2: Variables extracted from shortlisted articles.

Extracted variables	
General Article Information	● Author name<br>● Year published<br>● Years patients recruited from<br>● Country of study<br>● Sample size
Patient Characteristics	● Sex<br>● Age<br>● Body mass index<br>● Comorbidities<br>● Body surface area<br>● Performance status<br>● Selection criteria<br>● Paraneoplastic syndromes
Tumor Characteristics	● Resection status of prior surgery<br>● Tumor histology<br>● Side of pleural metastasis<br>● Lung infiltration<br>● Chest well infiltration<br>● Tumor stage<br>● Prior therapies<br>● WHO classification

Intraoperative Details	● Operative iatrogenic injuries<br>● Surgical technique and Operative approach<br>● Concurrent pneumonectomy<br>● Pericardium opened<br>● Anesthesia technique<br>● Extubation in the OR<br>● Resection status of present surgery<br>● HITHOC technique<br>● Preoperative hydration<br>● Mediastinal lymph nodes dissected<br>● Diaphragm dissected<br>● Chest wall resection<br>● HITHOC agents<br>● HITHOC dose<br>● HITHOC duration<br>● Intraoperative blood transfusion<br>● Intraoperative blood loss<br>● HITHOC temperature<br>● HITHOC perfusion rate<br>● HITHOC perfusion volume<br>● Duration of surgery<br>● Additional planned adjuvant after HITHOC<br>● Patient core temperature<br>● Patient respiratory rate<br>● Patient heart rate

Outcomes	● HITHOC-related toxicity<br>● Other complications<br>● Length of hospital stay<br>● Length of ICU stay<br>● Oncological outcomes<br>● Surgical outcomes<br>● Recurrence<br>● Site of recurrence/progression<br>● Time to recurrence<br>● Re-operation necessary after HITHOC<br>● Additional unplanned adjuvant after HITHOC<br>● Second HITHOC after first HITHOC<br>● Duration of follow-up<br>● Interval of follow-up

Supplementary Section S3: Risk of bias of included studies

Risk of bias of included studies

* The exposure measures (independent variables) were considered to be clearly defined if the study reported agent, dose and duration of HITHOC.

Study	Q1	Q2	Q3	Q4	Q5	Q6	Q7	Q8	Q9 *	Q10	Q11	Q12	Q13	Q14	Overall (%)
**Ambrogi et al. (2016)**	Yes	Yes	N/A	Yes	No	No	Yes	No	Yes	N/A	Yes	N/A	Yes	No	7/11 (63.6%)
**Aprile et al. (2020)**	Yes	Yes	N/A	Yes	No	No	Yes	No	Yes	N/A	Yes	N/A	Yes	Yes	8/11 (72.7%)
**Bree et al. (2022)**	Yes	Yes	N/A	Yes	No	No	Yes	No	Yes	N/A	Yes	N/A	Yes	No	7/11 (63.6%)
**Chappuy et al. (2022)**	Yes	Yes	N/A	Yes	No	No	Yes	No	Yes	N/A	Yes	N/A	Yes	No	7/11 (63.6%)
**Dolan et al. (2022)**	Yes	Yes	N/A	Yes	No	No	Yes	No	Yes	N/A	Yes	N/A	Yes	No	7/11 (63.6%)
**Klotz et al. (2021)**	Yes	Yes	N/A	Yes	No	No	Yes	No	Yes	N/A	Yes	N/A	Yes	No	7/11 (63.6%)
**Kodama et al. (2013)**	Yes	Yes	N/A	Yes	No	No	Yes	No	Yes	N/A	Yes	N/A	Yes	No	7/11 (63.6%)
**Kumar et al. (2021)**	Yes	Yes	N/A	Yes	No	No	Yes	No	Yes	N/A	Yes	N/A	Yes	No	7/11 (63.6%)
**Markowiak et al. (2021)**	Yes	Yes	N/A	Yes	No	No	Yes	No	Yes	N/A	Yes	N/A	Yes	No	7/11 (63.6%)
**Maury et al. (2017)**	Yes	Yes	N/A	Yes	No	No	Yes	No	Yes	N/A	Yes	N/A	Yes	No	7/11 (63.6%)
**Miller et al. (2023)**	Yes	Yes	Yes	Yes	No	No	Yes	No	Yes	N/A	Yes	N/A	Yes	No	8/12 (66.6%)
**Monneuse et al. (2003)**	Yes	Yes	Yes	Yes	No	No	Yes	No	Yes	N/A	Yes	N/A	Yes	No	8/12 (66.6%)
**Patel et al. (2018)**	Yes	Yes	N/A	Yes	No	No	No	No	Yes	N/A	Yes	N/A	Yes	No	6/11 (54.5%)
**Refaely et al. (2011)**	Yes	Yes	N/A	Yes	No	No	Yes	No	Yes	N/A	Yes	N/A	Yes	No	7/11 (63.6%)
**Ried et al. (2023)**	Yes	Yes	N/A	Yes	No	No	Yes	Yes	Yes	N/A	Yes	N/A	Yes	Yes	9/11 (81.8%)
**Yellin et al. (2013)**	Yes	Yes	N/A	Yes	No	No	Yes	No	Yes	N/A	Yes	N/A	Yes	No	7/11 (63.6%)

**Yu et al. (2013)**	Yes	Yes	Yes	Yes	No	No	Yes	No	Yes	N/A	Yes	N/A	Ye s	No	8/12 (66.6%)
